# 3-(4-Bromo­phen­yl)quinazolin-4(3*H*)-one

**DOI:** 10.1107/S1600536811040736

**Published:** 2011-10-12

**Authors:** T. Srinivasan, S. Suhitha, M. Gnana Ruba Priya, K. Girija, N. Ravi Chandran, D. Velmurugan

**Affiliations:** a Centre of Advanced Study in Crystallography and Biophysics, University of Madras, Guindy Campus, Chennai 600 025, India; b Department of Chemistry, Sastra University, Thanjavur 613 402, India; c Mother Theresa Postgraduate & Health Science, Puducherry 605 006, India; d Department of Carism, Sastra University, Thanjavur 613 402, India

## Abstract

In the title compound, C_14_H_9_BrN_2_O, the quinazoline unit is essentially planar, with a mean deviation of 0.058 (2) Å from the least-squares plane defined by the ten constituent ring atoms. The dihedral angle between the mean plane of the quinazoline ring system and the 4-bromo­phenyl ring is 47.6 (1)°. In the crystal, mol­ecules are linked by inter­molecular C—H⋯N and C—H⋯O hydrogen bonds, forming infinite chains of alternating *R*_2_^2^(6) dimers and *R*_2_^2^(14) ring motifs.

## Related literature

For the synthesis of the title compound, see: Priya, Zulykama *et al.* (2011 ▶). For a related structure, see: Priya, Srinivasan *et al.* (2011 ▶). For the biological activity of quinazoline derivatives, see: Wolfe *et al.*(1990 ▶); Tereshima *et al.* (1995 ▶); Pandeya *et al.* (1999 ▶). For graph-set notation, see: Bernstein *et al.* (1995 ▶).
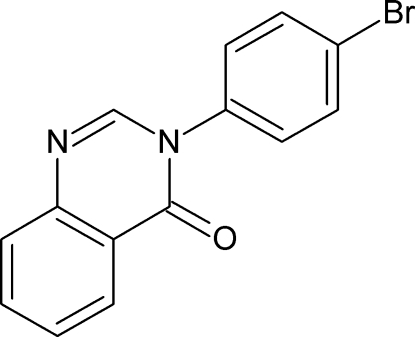


## Experimental

### 

#### Crystal data

C_14_H_9_BrN_2_O
*M* *_r_* = 301.14
Monoclinic, 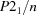

*a* = 16.961 (3) Å
*b* = 3.9530 (8) Å
*c* = 17.698 (3) Å
β = 93.168 (11)°
*V* = 1184.8 (4) Å^3^
*Z* = 4
Mo *K*α radiation
μ = 3.46 mm^−1^
*T* = 293 K
0.20 × 0.20 × 0.20 mm

#### Data collection

Bruker SMART APEXII area-detector diffractometer
10371 measured reflections
2840 independent reflections
1772 reflections with *I* > 2σ(*I*)
*R*_int_ = 0.050

#### Refinement

*R*[*F* ^2^ > 2σ(*F* ^2^)] = 0.036
*wR*(*F* ^2^) = 0.094
*S* = 1.01
2840 reflections
163 parameters
H-atom parameters constrained
Δρ_max_ = 0.34 e Å^−3^
Δρ_min_ = −0.54 e Å^−3^

### 

Data collection: *APEX2* (Bruker, 2008 ▶); cell refinement: *SAINT* (Bruker, 2008 ▶); data reduction: *SAINT*; program(s) used to solve structure: *SHELXS97* (Sheldrick, 2008 ▶); program(s) used to refine structure: *SHELXL97* (Sheldrick, 2008 ▶); molecular graphics: *ORTEP-3* (Farrugia, 1997 ▶); software used to prepare material for publication: *SHELXL97* and *PLATON* (Spek, 2009 ▶).

## Supplementary Material

Crystal structure: contains datablock(s) global, I. DOI: 10.1107/S1600536811040736/im2320sup1.cif

Structure factors: contains datablock(s) I. DOI: 10.1107/S1600536811040736/im2320Isup2.hkl

Supplementary material file. DOI: 10.1107/S1600536811040736/im2320Isup3.cml

Additional supplementary materials:  crystallographic information; 3D view; checkCIF report

## Figures and Tables

**Table 1:** Hydrogen-bond geometry (Å, °)

*D*—H⋯*A*	*D*—H	H⋯*A*	*D*⋯*A*	*D*—H⋯*A*
C8—H8⋯N1^i^	0.93	2.48	3.286 (4)	146
C11—H11⋯O1^ii^	0.93	2.32	3.224 (4)	165

Symmetry codes: (i) 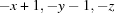; (ii) 
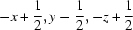
.

**Fig. 1.**
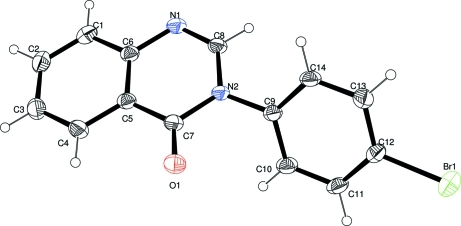
Molecular structure of the title compound. Displacement ellipsoids are drawn at the 30% probability level. H atoms are presented as a small spheres of arbitrary radius.

**Fig. 2.**
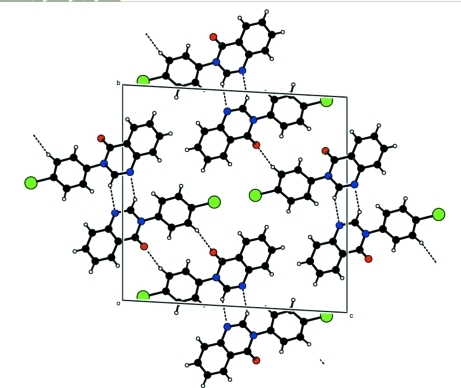
View of the C–H···N and C–H···O hydrogen bonds (dotted lines) in the crystal structure of the title compound. [Symmetry codes: (i) - x + 1, - y -1, - z; (ii) - x +1/2, y - 1/2, - z + 1/2.]

## supplementary crystallographic information

### Comment

4(3H)-Quinazolinones are an important class of fused heterocycles with a wide range of biological activities such as anti-cancer (Wolfe *et al.*,1990), anti-inflammatory (Tereshima *et al.*,1995) and anti-HIV (Pandeya *et al.*, 1999). In addition to that, quinazolinones exibit anti-bacterial and anti-fungal activities (Priya, Zulykama *et al.*, 2011).

In title molecule (Fig. 1), the quinazoline unit is essentially planar, with a mean deviation of 0.058 (2) Å from the least square plane defined by the ten constituent atoms. The dihedral angle formed by the 4-bromophenyl ring and the mean plane of the quinazoline fragment is 47.6 (1)° . In the crystal packing, molecules are linked by intermolecular C–H···N and C–H···O hydrogen bonds (Table 1). These hydrogen bonds are forming infinite chains of alternating R_2_^2^(6) dimer and R_2_^2^(14) ring motifs (Bernstein *et al.*, 1995) as shown in Fig. 2.

### Experimental

To an ice-cold solution of 2.8 ml POCl_3_ in 5 ml DMF was added anthranilic acid (2 g, 0.0146 mole) and stirred for 5-10 min until TLC indicated the disappearance of anthranilic acid. The reaction mixture was then treated with an equimolar amount of *p*-bromo-aniline (2.511 g) and supported on anhydrous sodium sulfate (five times the weight of anthranilic acid) and exposed to microwave (BPL company) irradiation (600 W) for 2-4 min with 30 sec pulse. The reaction mixture was quenched with water (50 ml) and extracted with ethyl acetate (2 x 50 ml). The organic layer was dried over anhydrous sodium sulfate, concentrated and purified by silica gel column chromatography (60-20 mesh) using hexane/EtOAc (7.5 : 2.5) as eluent to yield the pure product (yield: 4,397 g, 84%). Single crystals suitable for X-ray diffraction were prepared by slow evaporation of a solution of the title compound in methanol at room temperature.

### Refinement

Hydrogen atoms were placed in calculated positions with C–H = 0.93 Å and refined using a riding model with fixed a isotropic displacement parameter of *U*_iso_(H) = 1.2 *U*_eq_(C).

### Crystal data

**Table:**

C_14_H_9_BrN_2_O	*F*(000) = 600
*M**_r_* = 301.14	*D*_x_ = 1.688 Mg m^−^^3^
Monoclinic, *P*2_1_/*n*	Mo *K*α radiation, λ = 0.71073 Å
Hall symbol: -P 2yn	Cell parameters from 2840 reflections
*a* = 16.961 (3) Å	θ = 1.6–28.3°
*b* = 3.9530 (8) Å	µ = 3.46 mm^−^^1^
*c* = 17.698 (3) Å	*T* = 293 K
β = 93.168 (11)°	Block, colourless
*V* = 1184.8 (4) Å^3^	0.20 × 0.20 × 0.20 mm
*Z* = 4	

### Data collection

**Table:**

Bruker SMART APEXII area-detector diffractometer	1772 reflections with *I* > 2σ(*I*)
Radiation source: fine-focus sealed tube	*R*_int_ = 0.050
graphite	θ_max_ = 28.3°, θ_min_ = 1.6°
ω and φ scans	*h* = −22→14
10371 measured reflections	*k* = −4→5
2840 independent reflections	*l* = −23→23

### Refinement

**Table:**

Refinement on *F*^2^	Primary atom site location: structure-invariant direct methods
Least-squares matrix: full	Secondary atom site location: difference Fourier map
*R*[*F*^2^ > 2σ(*F*^2^)] = 0.036	Hydrogen site location: inferred from neighbouring sites
*wR*(*F*^2^) = 0.094	H-atom parameters constrained
*S* = 1.01	*w* = 1/[σ^2^(*F*_o_^2^) + (0.0381*P*)^2^ + 0.2934*P*] where *P* = (*F*_o_^2^ + 2*F*_c_^2^)/3
2840 reflections	(Δ/σ)_max_ = 0.001
163 parameters	Δρ_max_ = 0.34 e Å^−^^3^
0 restraints	Δρ_min_ = −0.54 e Å^−^^3^

### Special details

**Table:**

Geometry. All esds (except the esd in the dihedral angle between two l.s. planes) are estimated using the full covariance matrix. The cell esds are taken into account individually in the estimation of esds in distances, angles and torsion angles; correlations between esds in cell parameters are only used when they are defined by crystal symmetry. An approximate (isotropic) treatment of cell esds is used for estimating esds involving l.s. planes.
Refinement. Refinement of F^2^ against ALL reflections. The weighted R-factor wR and goodness of fit S are based on F^2^, conventional R-factors R are based on F, with F set to zero for negative F^2^. The threshold expression of F^2^ > 2sigma(F^2^) is used only for calculating R-factors(gt) etc. and is not relevant to the choice of reflections for refinement. R-factors based on F^2^ are statistically about twice as large as those based on F, and R- factors based on ALL data will be even larger.

### Fractional atomic coordinates and isotropic or equivalent isotropic displacement parameters (Å^2^)

**Table:**

	*x*	*y*	*z*	*U*_iso_*/*U*_eq_	
C1	0.30495 (19)	−0.4078 (7)	−0.13542 (16)	0.0457 (8)	
H1	0.3413	−0.5172	−0.1644	0.055*	
C2	0.22902 (19)	−0.3495 (8)	−0.16454 (17)	0.0494 (8)	
H2	0.2143	−0.4201	−0.2134	0.059*	
C3	0.17450 (19)	−0.1869 (8)	−0.12177 (18)	0.0500 (8)	
H3	0.1233	−0.1520	−0.1418	0.060*	
C4	0.19570 (17)	−0.0771 (8)	−0.04997 (17)	0.0445 (7)	
H4	0.1591	0.0340	−0.0216	0.053*	
C5	0.27242 (16)	−0.1327 (7)	−0.01962 (15)	0.0354 (7)	
C6	0.32663 (17)	−0.3015 (7)	−0.06239 (15)	0.0360 (6)	
N2	0.37065 (13)	−0.1237 (5)	0.08283 (12)	0.0338 (5)	
C9	0.39734 (16)	−0.0577 (7)	0.15992 (14)	0.0340 (6)	
C10	0.35023 (17)	−0.1470 (7)	0.21832 (16)	0.0424 (7)	
H10	0.3018	−0.2522	0.2077	0.051*	
C11	0.37520 (18)	−0.0797 (7)	0.29172 (16)	0.0430 (7)	
H11	0.3434	−0.1357	0.3310	0.052*	
C12	0.44731 (17)	0.0707 (7)	0.30707 (15)	0.0383 (7)	
C13	0.49575 (17)	0.1529 (7)	0.24960 (16)	0.0440 (7)	
H13	0.5449	0.2506	0.2607	0.053*	
C14	0.47056 (16)	0.0889 (7)	0.17584 (16)	0.0397 (7)	
H14	0.5026	0.1440	0.1367	0.048*	
C7	0.29483 (16)	−0.0184 (8)	0.05649 (15)	0.0392 (7)	
C8	0.41999 (17)	−0.2860 (7)	0.03558 (16)	0.0380 (7)	
H8	0.4704	−0.3381	0.0556	0.046*	
O1	0.25479 (13)	0.1572 (6)	0.09574 (12)	0.0599 (6)	
N1	0.40348 (14)	−0.3727 (6)	−0.03334 (13)	0.0411 (6)	
Br1	0.47919 (2)	0.17499 (9)	0.408526 (17)	0.05840 (16)	

### Atomic displacement parameters (Å^2^)

**Table:**

	*U*^11^	*U*^22^	*U*^33^	*U*^12^	*U*^13^	*U*^23^
C1	0.056 (2)	0.0462 (19)	0.0358 (15)	0.0011 (15)	0.0107 (14)	−0.0001 (13)
C2	0.057 (2)	0.053 (2)	0.0376 (15)	−0.0083 (17)	−0.0049 (15)	0.0042 (14)
C3	0.0436 (18)	0.056 (2)	0.0494 (18)	−0.0026 (16)	−0.0041 (15)	0.0095 (16)
C4	0.0351 (17)	0.0494 (19)	0.0493 (17)	0.0077 (14)	0.0058 (14)	0.0051 (14)
C5	0.0356 (16)	0.0347 (17)	0.0368 (14)	0.0037 (13)	0.0094 (12)	0.0068 (12)
C6	0.0361 (16)	0.0359 (15)	0.0367 (14)	0.0006 (13)	0.0081 (12)	0.0063 (12)
N2	0.0301 (12)	0.0408 (14)	0.0314 (11)	0.0064 (11)	0.0090 (9)	0.0002 (10)
C9	0.0335 (16)	0.0367 (16)	0.0325 (13)	0.0026 (13)	0.0077 (12)	0.0035 (12)
C10	0.0390 (17)	0.0477 (19)	0.0416 (15)	−0.0047 (14)	0.0122 (13)	0.0043 (14)
C11	0.0467 (18)	0.0506 (19)	0.0331 (14)	−0.0002 (15)	0.0153 (13)	0.0068 (13)
C12	0.0453 (18)	0.0410 (16)	0.0291 (13)	0.0023 (14)	0.0050 (12)	0.0026 (12)
C13	0.0379 (16)	0.0505 (19)	0.0434 (16)	−0.0070 (15)	0.0018 (13)	0.0042 (14)
C14	0.0341 (16)	0.0474 (19)	0.0389 (15)	−0.0011 (14)	0.0131 (12)	0.0086 (13)
C7	0.0316 (16)	0.0478 (18)	0.0390 (15)	0.0039 (15)	0.0083 (12)	0.0052 (14)
C8	0.0304 (15)	0.0447 (17)	0.0397 (15)	0.0077 (13)	0.0096 (12)	0.0039 (13)
O1	0.0482 (13)	0.0870 (17)	0.0452 (12)	0.0302 (12)	0.0080 (10)	−0.0133 (12)
N1	0.0372 (14)	0.0494 (16)	0.0377 (13)	0.0062 (12)	0.0117 (11)	−0.0007 (11)
Br1	0.0734 (3)	0.0652 (3)	0.03636 (18)	−0.00188 (19)	0.00039 (15)	−0.00357 (15)

### Geometric parameters (Å, °)

**Table:**

C1—C2	1.380 (4)	C9—C14	1.385 (4)
C1—C6	1.389 (4)	C9—C10	1.387 (4)
C1—H1	0.9300	C10—C11	1.370 (4)
C2—C3	1.385 (4)	C10—H10	0.9300
C2—H2	0.9300	C11—C12	1.373 (4)
C3—C4	1.372 (4)	C11—H11	0.9300
C3—H3	0.9300	C12—C13	1.381 (4)
C4—C5	1.398 (4)	C12—Br1	1.892 (3)
C4—H4	0.9300	C13—C14	1.374 (4)
C5—C6	1.393 (4)	C13—H13	0.9300
C5—C7	1.451 (4)	C14—H14	0.9300
C6—N1	1.403 (4)	C7—O1	1.216 (3)
N2—C8	1.374 (3)	C8—N1	1.283 (4)
N2—C7	1.406 (3)	C8—H8	0.9300
N2—C9	1.437 (3)		
			
C2—C1—C6	119.4 (3)	C10—C9—N2	119.8 (2)
C2—C1—H1	120.3	C11—C10—C9	119.8 (3)
C6—C1—H1	120.3	C11—C10—H10	120.1
C1—C2—C3	120.7 (3)	C9—C10—H10	120.1
C1—C2—H2	119.6	C10—C11—C12	119.8 (3)
C3—C2—H2	119.6	C10—C11—H11	120.1
C4—C3—C2	120.3 (3)	C12—C11—H11	120.1
C4—C3—H3	119.9	C11—C12—C13	121.0 (3)
C2—C3—H3	119.9	C11—C12—Br1	119.1 (2)
C3—C4—C5	119.8 (3)	C13—C12—Br1	119.8 (2)
C3—C4—H4	120.1	C14—C13—C12	119.3 (3)
C5—C4—H4	120.1	C14—C13—H13	120.3
C6—C5—C4	119.7 (3)	C12—C13—H13	120.3
C6—C5—C7	120.4 (2)	C13—C14—C9	119.9 (2)
C4—C5—C7	119.9 (2)	C13—C14—H14	120.1
C1—C6—C5	120.1 (3)	C9—C14—H14	120.1
C1—C6—N1	118.2 (3)	O1—C7—N2	120.6 (3)
C5—C6—N1	121.6 (2)	O1—C7—C5	125.6 (3)
C8—N2—C7	120.9 (2)	N2—C7—C5	113.8 (2)
C8—N2—C9	119.5 (2)	N1—C8—N2	126.5 (3)
C7—N2—C9	119.7 (2)	N1—C8—H8	116.7
C14—C9—C10	120.1 (3)	N2—C8—H8	116.7
C14—C9—N2	120.1 (2)	C8—N1—C6	116.4 (2)
			
C6—C1—C2—C3	0.0 (4)	C10—C11—C12—Br1	177.9 (2)
C1—C2—C3—C4	0.9 (5)	C11—C12—C13—C14	1.3 (5)
C2—C3—C4—C5	−0.6 (5)	Br1—C12—C13—C14	−177.3 (2)
C3—C4—C5—C6	−0.5 (4)	C12—C13—C14—C9	−0.2 (4)
C3—C4—C5—C7	−179.7 (3)	C10—C9—C14—C13	−1.5 (4)
C2—C1—C6—C5	−1.1 (4)	N2—C9—C14—C13	179.7 (3)
C2—C1—C6—N1	178.2 (2)	C8—N2—C7—O1	−171.8 (3)
C4—C5—C6—C1	1.3 (4)	C9—N2—C7—O1	7.5 (4)
C7—C5—C6—C1	−179.5 (3)	C8—N2—C7—C5	6.9 (4)
C4—C5—C6—N1	−177.9 (2)	C9—N2—C7—C5	−173.8 (2)
C7—C5—C6—N1	1.3 (4)	C6—C5—C7—O1	172.8 (3)
C8—N2—C9—C14	48.8 (4)	C4—C5—C7—O1	−8.0 (4)
C7—N2—C9—C14	−130.5 (3)	C6—C5—C7—N2	−5.9 (4)
C8—N2—C9—C10	−130.0 (3)	C4—C5—C7—N2	173.3 (2)
C7—N2—C9—C10	50.7 (4)	C7—N2—C8—N1	−3.4 (4)
C14—C9—C10—C11	2.1 (4)	C9—N2—C8—N1	177.3 (3)
N2—C9—C10—C11	−179.1 (3)	N2—C8—N1—C6	−1.7 (4)
C9—C10—C11—C12	−1.1 (5)	C1—C6—N1—C8	−176.6 (3)
C10—C11—C12—C13	−0.6 (5)	C5—C6—N1—C8	2.7 (4)

### Hydrogen-bond geometry (Å, °)

**Table:**

*D*—H···*A*	*D*—H	H···*A*	*D*···*A*	*D*—H···*A*
C8—H8···N1^i^	0.93	2.48	3.286 (4)	146.
C11—H11···O1^ii^	0.93	2.32	3.224 (4)	165.

Symmetry codes: (i) −*x*+1, −*y*−1, −*z*; (ii) −*x*+1/2, *y*−1/2, −*z*+1/2.
